# Melatonin Confers Plant Cadmium Tolerance: An Update

**DOI:** 10.3390/ijms222111704

**Published:** 2021-10-28

**Authors:** Quan Gu, Chuyan Wang, Qingqing Xiao, Ziping Chen, Yi Han

**Affiliations:** 1 School of Biology, Food and Environment, Hefei University, Hefei 230601, China; guq@hfuu.edu.cn (Q.G.); wangchuyan19@163.com (C.W.); xiaoqq@hfuu.edu.cn (Q.X.); 2 State Key Laboratory of Tea Plant Biology and Utilization, Anhui Agricultural University, Hefei 230036, China; 3 National Engineering Laboratory of Crop Stress Resistence Breeding, School of Life Sciences, Anhui Agricultural University, Hefei 230036, China

**Keywords:** antioxidant defense systems, Cd stress, hydrogen sulfide, melatonin, oxidative stress, transportation and sequestration

## Abstract

Cadmium (Cd) is one of the most injurious heavy metals, affecting plant growth and development. Melatonin (*N*-acetyl-5-methoxytryptamine) was discovered in plants in 1995, and it is since known to act as a multifunctional molecule to alleviate abiotic and biotic stresses, especially Cd stress. Endogenously triggered or exogenously applied melatonin re-establishes the redox homeostasis by the improvement of the antioxidant defense system. It can also affect the Cd transportation and sequestration by regulating the transcripts of genes related to the major metal transport system, as well as the increase in glutathione (GSH) and phytochelatins (PCs). Melatonin activates several downstream signals, such as nitric oxide (NO), hydrogen peroxide (H_2_O_2_), and salicylic acid (SA), which are required for plant Cd tolerance. Similar to the physiological functions of NO, hydrogen sulfide (H_2_S) is also involved in the abiotic stress-related processes in plants. Moreover, exogenous melatonin induces H_2_S generation in plants under salinity or heat stress. However, the involvement of H_2_S action in melatonin-induced Cd tolerance is still largely unknown. In this review, we summarize the progresses in various physiological and molecular mechanisms regulated by melatonin in plants under Cd stress. The complex interactions between melatonin and H_2_S in acquisition of Cd stress tolerance are also discussed.

## 1. Introduction

Heavy metal pollution is the most widespread contamination resulting from anthropogenic activities in the world [1]. It has raised concerns about its various harmful risks to human health via the metal transfer along the food chain [2]. Among the heavy metals, cadmium (Cd) is a toxic element and poses a hazardous impact to living organisms, such as renal tubular dysfunction and bone disease [3]. In plants, Cd disturbs a range of important biochemical, morphological, physiological, and molecular processes, thus resulting in chlorosis and shunted growth [4,5]. Cd stress deceases the chlorophyll content, net photosynthetic rate, stomatal conductance, intracellular CO_2_ concentration, and transpiration rate [4,5,6]. Cd stress induces the excess accumulation of reactive oxygen species (ROS), mainly due to the imbalance between ROS generation and scavenging [7,8]. Increased concentrations of ROS further induce the lipid peroxidation and oxidative damage, destructing plant membranes, macromolecules, and organelles [7,8]. Additionally, excessive bioaccumulation of Cd in plants inhibits Fe and Zn uptake, and disrupts the uptake and transport of K, Ca, Mg, P, and Mn [9]. In response to Cd stress, plants have evolved the complex biochemical and molecular mechanisms that modulate ROS homeostasis and Cd compartmentation and chelation [7,10,11,12]. Plant hormones (ethylene, salicylic acid (SA), abscisic acid (ABA), jasmonic acid (JA), auxin, brassinosteroids (BRs), and strigolactones (SLs)) and signaling molecules (nitric oxide (NO), carbon monoxide (CO), hydrogen sulfide (H_2_S), and Ca^2+^) are involved in plant response to Cd stress [13,14]. Moreover, recent studies have reported that melatonin acts as a master regulator in plant Cd tolerance.

Melatonin (*N*-acetyl-5-methoxytryptamine) was discovered in plants in 1995, and it is since known to act as a pleiotropic molecule to participate in multiple physiological processes, such as plant growth and development, and protection against abiotic and biotic stresses [15,16]. In recent years, numerous studies have focused on the protective role of melatonin against Cd stress in plants [17]. Application of exogenous melatonin increased photosynthetic pigments, and improved relative water content and stomatal conductance in mallow plants upon Cd stress [18]. Many results showed that melatonin could re-establish redox homeostasis by certain enzymatic and non-enzymatic antioxidant defense systems to alleviate Cd-induced oxidative stress [19,20]. In addition, melatonin decreased Cd accumulation via regulating the transcripts of several heavy metal transporter genes to restrict Cd influx, and promote Cd efflux and chelation [19,21]. Moreover, NO and hydrogen peroxide (H_2_O_2_) signaling, microRNAs, heat shock factor HsfA1a and flavonoids may be involved in melatonin-mediated Cd tolerance in plants [19,22,23,24,25].

Apart from NO and H_2_O_2_, H_2_S may also function as a signaling molecule in numerous processes of plants [26,27,28]. It is produced from the degradation of L-cysteine by L-cysteine desulfhydrase (L-CDes), which is encoded by L-cysteine desulfhydrase (*LCD*), D-cysteine desulfhydrase (*DCD*), and L-cysteine desulfhydrase1 (*DES1*) [29,30]. Exogenous application of H_2_S donors regulated plant growth, and conferred tolerance to salinity, heavy metal, heat, and drought stress among others [27,31,32]. H_2_S enhanced photosynthesis and antioxidant enzyme activity, and up-regulated the transcripts of PC genes to alleviate Cd stress in tobacco [33]. Moreover, H_2_S homeostasis and L-cysteine desulfhydrase activity were involved in melatonin-modulated salt stress tolerance in tomato and cucumber seedlings [31,34,35]. Crosstalk of melatonin and H_2_S in alleviating heat stress was also suggested in wheat [36]. However, the involvement of H_2_S action in melatonin-mediated abiotic stress tolerance is still largely unknown, especially in Cd stress.

Over the past several years, numerous studies focusing on the role of melatonin in alleviating Cd stress have been steadily increasing in plants. Here, we systematically review and highlight the advanced developments which explore the melatonin-mediated Cd tolerance. For a better understanding of this topic, we also propose and discuss the future studies on the complex interactions between melatonin and H_2_S during Cd stress.

## 2. Role of Melatonin in Plant Abiotic Stress Responses

### 2.1. Melatonin Biosynthesis and Catabolism

**Figure 1:**
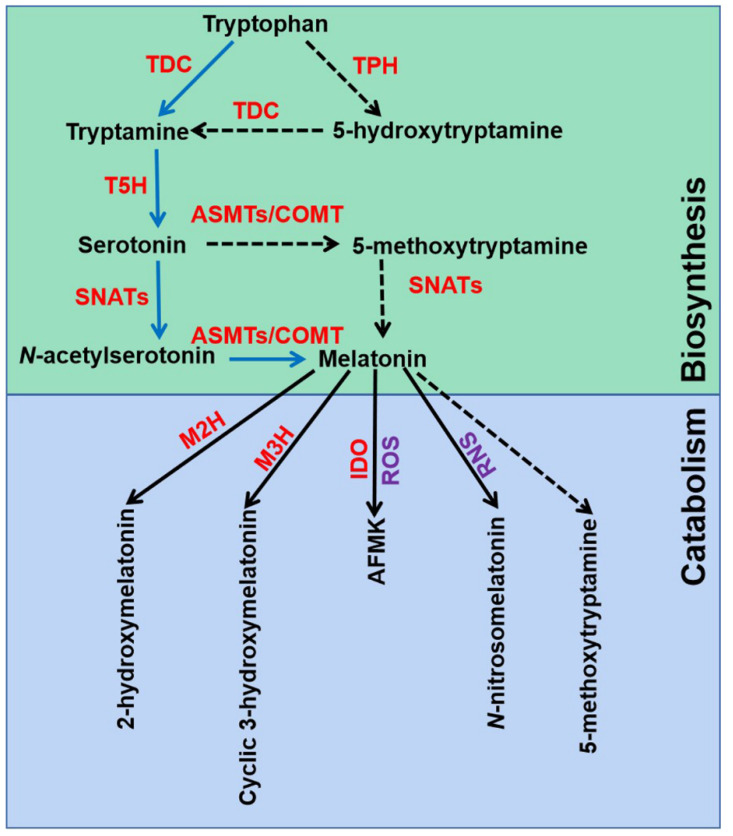
Melatonin biosynthesis and metabolic pathways in plants. TDC, tryptophan decarboxylase; T5H, tryptamine 5-hydroxylase; TPH, tryptophan hydroxylase; SNATs, serotonin N-acetyltransferases; ASMTs, N-acetylserotonin-O-methyltransferases; COMT, caffeic acid O-methyltransferase; M2H, melatonin 2-hydroxylase; M3H, melatonin 3-hydroxylase; IDO, indoleamine 2,3-dioxygenase; AFMK, *N*^1^-acetyl-*N*^2^-formyl-5-methoxykynuramine; ROS, reactive oxygen species; RNS, reactive nitrogen species. The green box indicates melatonin biosynthesis pathways, and blue box indicates melatonin metabolic pathways.

The melatonin metabolic pathway in plants contains two major parts: biosynthesis and catabolism (Figure 1). Melatonin was discovered and confirmed by an isotope tracer study of St. John’s wort (*Hypericum perforatum* L. cv. Anthos) seedlings [15,37]. It was found that melatonin is synthesized via four continual enzymatic reactions from tryptophan, requiring at least six enzymes: tryptophan decarboxylase (TDC), tryptophan hydroxylase (TPH), tryptamine 5-hydroxylase (T5H), *N*-acetylserotonin methyltransferase (ASMT), and serotonin *N*-acetyltransferase (SNAT) [17]. T5H-catalyzed hydroxylation of tryptamine is an important step of melatonin biosynthesis in rice (*Oryza sativa*) [38]. In animals, serotonin is initially acetylated to form *N*-acetylserotonin, and then *O*-methylated to form melatonin (named NM pathway) [39]. It has also been found that serotonin is *O*-methylated to form 5-methoxytryptamine, and then acetylated to form melatonin (named MN pathway) [39]. Both NM and MN pathways exist in plants [40].

Melatonin can be degraded by two distinct routes: non-enzymatic and enzymatic transformations [17]. Transgenic tomato (*Solanum lycopersicum*) plants expressing the gene encoding indoleamine 2,3-dioxygenase (IDO) in rice showed reduced melatonin levels [41]. Thus, the pathway that melatonin converts to *N*^1^-acetyl-*N*^2^-formyl-5-methoxykynuramine (AFMK) exists in plants. Tan and Reiter speculated that AFMK is the product of melatonin interaction with ROS, which generated during photosynthesis [39]. This might reflect the important role of melatonin in detoxifying ROS accumulation. In addition, melatonin hydroxylation metabolites, 2-hydroxymelatonin (2-OHMel) and cyclic 3-hydroxymelatonin (c3-OHMel), have been identified in plants. Their formation is attributed to melatonin 2-hydroxylase (M2H) and melatonin 3-hydroxylase (M3H), respectively [42,43,44]. Singh et al. suggested that *N*-nitrosomelatonin (NOmela) likely served as a nitric oxide (NO) carrier that participated in the redox signal transduction [45]. Nevertheless, Mukherjee considered that NOmela served as an intracellular NO reserve in plants was questionable due to its sensitive and unstable characteristics [46]. The processes of NOmela formation and transport are not fully understood and should be thoroughly investigated. In addition, whether 5-methoxytryptamine (5-MT) formed by melatonin deacetylation is of physiological importance remains to be investigated in plants.

### 2.2. Melatonin Acts as a Master Regulator in Plant Abiotic Stress

**Figure 2:**
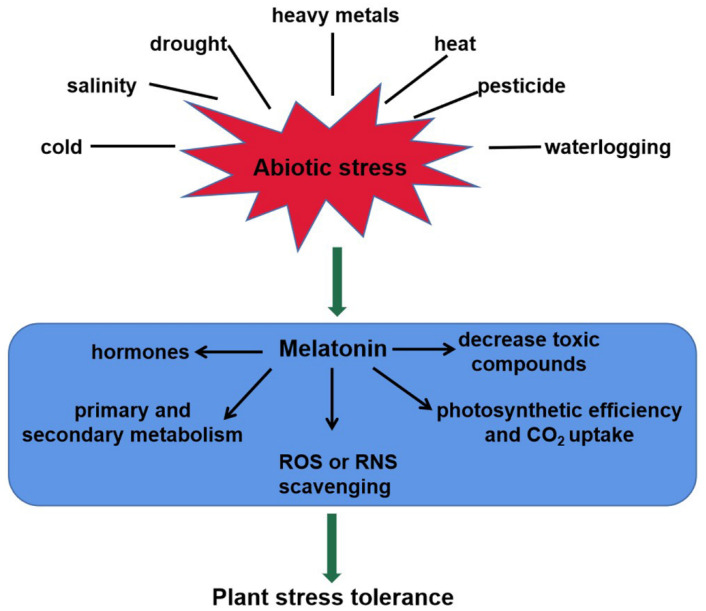
The roles of melatonin in plant tolerance to abiotic stress. Melatonin content of plants increases significantly in responses to abiotic stresses, such as heavy metals, salinity, drought, heat, cold, waterlogging, and pesticides. It confers plant tolerance via multiple mechanisms, including ROS or RNS scavenging, toxic compounds decrease, photosynthetic efficiency increase, interaction with hormones, and secondary metabolite biosynthesis. ROS, reactive oxygen species; RNS, reactive nitrogen species.

As a master regulator, melatonin plays important roles in plant tolerance to abiotic stresses, such as heavy metals, drought, salinity, cold, heat, waterlogging, and pesticides [19,47,48,49,50,51,52]. This review shows schematically the melatonin-mediated responses to abiotic stresses in plants (Figure 2). Melatonin levels are strongly induced by the above unfavorable conditions. For instance, endogenous melatonin level in *Arabidopsis* wild-type plants was increased in response to salt stress [47]. Loss-of-function mutation *atsnat* in the *AtSNAT* gene showed lower endogenous melatonin content and sensitivity to salinity stress [47]. Cold stress induced melatonin accumulation by upregulating the relative expression of *ClASMT* in watermelon plants [49]. In tomato seedlings, Cd stress induced *COMT1* expression, and thereby improved the accumulation of melatonin [22]. Transcription factor heat shock factor A1a (HsfA1a) bound to the *COMT1* gene promoter and activated the transcription of *COMT1* gene under Cd stress [22]. However, the post-translational regulation of melatonin biosynthesis genes and modification of related proteins still remains largely unknown and should be elucidated in future.

Melatonin confers plant tolerance via multiple mechanisms, including photosynthetic efficiency increase, ROS or RNS scavenging, toxic compounds decrease, interaction with hormones, and secondary metabolite biosynthesis (Figure 2). Melatonin stimulated stomatal conductance and improved photosynthesis, thus enhancing tolerance to water-deficient stress in grape cuttings [53]. Another fact is that the photosynthetic efficiency was maximized by higher rates of CO_2_ assimilation and stomatal conductance after application of melatonin [54]. Several stresses can induce ROS or RNS accumulation, causing oxidative damage to plants [55]. In this case, melatonin re-establishes the redox balance via activating enzymatic antioxidant defense systems, as well as the ascorbate–glutathione (AsA-GSH) cycle [56]. In plants, the Salt-Overly Sensitive (SOS) pathway mediates ionic homeostasis and contributes to salinity tolerance [57]. This pathway comprises three crucial genes, Salt-Overly Sensitive1 (*SOS1*), Salt-Overly Sensitive2 (*SOS2*) and Salt-Overly Sensitive3 (*SOS3*), which function together to initiate transport of Na^+^ out of the cell, or activating other transporters, thus leading to the sequestration of Na^+^ in the vacuole [58]. Melatonin reduced ion toxicity and improved salinity tolerance via the SOS pathway [47]. ABA and H_2_O_2_/NO signaling transduction pathways were also modulated for plant tolerance in response to abiotic stress [47,48,56,59]. In addition, melatonin could increase primary and secondary metabolites including amino acids, organic acids and sugars, and thus improving plant cold tolerance [60].

## 3. Melatonin Improves Cd Tolerance in Plants

It has been found that Cd affects the ecosystem, causing stress and toxicity in plants. Melatonin acts as a key role in protecting plants from Cd stress. Table 1 summarizes that Cd treatment up-regulates the transcripts of melatonin biosynthesis genes, such as *TDC*, *T5H*, *SNAT*, *ASMT*, and *COMT* in *Arabidopsis thaliana*, *Oryza sativa* L., *Solanum lycopersicum*, *Triticum aestivum* L., *Nicotiana tabacum* L., and *Agaricus campestris* [59,61,62,63,64,65,66,67]. Therefore, melatonin contents are significantly increased. Notably, four *M2H* genes, involved in melatonin degradation, were also induced [65]. Byeon et al. suggested that both melatonin degradation and melatonin synthesis occurred in parallel, and 2-hydroxymelatonin of melatonin metabolite also acted as a signaling molecule in plant stress tolerance [65]. As melatonin catabolism is complicated, other pathways and the role of their metabolites should be investigated in plants under Cd stress.

Most studies showed that melatonin alleviated Cd-induced seedling growth inhibition, including the biomass (fresh weight and dry weight) and root length [19]. Melatonin improved the photosynthesis rate (Pn), transpiration rate (E), intracellular CO_2_ concentration and stomatal conductance (Gs) upon Cd stress in *Nicotiana tabacum* L. [6]. That melatonin enhanced stomatal opening and conductance capacity ultimately favored the photosynthesis in plants. Melatonin also prevented the degradation of the chlorophyll and carotenoid molecules in Chinese cabbage seedlings [68]. Similarly, application of melatonin improved chlorophyll and the maximum quantum efficiency of photosystem II (Fv/Fm) levels of wheat plants [20]. In chloroplasts, superoxide anion (O_2_**·**^−^) in photosystem I (PSI) is generated by two molecules of O_2_ with two electrons from photosystem II (PSII), and disproportionated to H_2_O_2_ catalyzed with superoxide dismutase (SOD) [69]. The better potential in melatonin treated plants under Cd stress can aid in chlorophyll protection, improve photosynthesis, and mediate redox homeostasis from oxidative damage.

### 3.1. Melatonin Activates Antioxidant Defense Systems in Response to Cd Stress

Cd stress induces ROS overproduction, containing H_2_O_2_, O_2_**·**^−^, hydroxyl radical (**·**OH), and singlet oxygen (^1^O_2_) [70]. These could be formed in photosynthetic cells, mitochondrial respiratory electron transport chain, respiratory processes, and nicotinamide-adenine dinucleotide phosphate (NADPH) oxidases in chloroplasts, mitochondria, peroxisomes, and plasma membrane, respectively [71]. Plants have evolved two antioxidant systems to relieve the ROS-triggered damages, including the enzymatic and non-enzymatic defense systems. Enzymatic defense systems including catalase (CAT), ascorbate peroxidase (APX), guaiacol peroxidase (POD), SOD, glutathione peroxidases (GPX), glutathione reductase (GR), dehydroascorbate reductase (DHAR), peroxiredoxins (PRX), thioredoxins (TRX), and glutaredoxins (GRX) are responsible for ROS scavenging [71]. The non-enzymatic systems, including ascorbate, GSH, flavonoid, anthocyanins, sugars, and carotenoids, are also essential for ROS elimination [71,72,73,74].

Melatonin protects plants by enhancing the ROS scavenging efficiency in response to Cd-induced oxidative stress. Application of exogenous melatonin significantly decreased H_2_O_2_, malondialdehyde (MDA), and O_2_**·**^−^ levels in the tomato leaves/roots under Cd stress [75]. Similar results were also observed in *Triticum aestivum* L., *Nicotiana tabacum* L., *Brassica napus* L., *Catharanthus roseus* (L.), *Malva parviflora*, *Fragaria x ananassa* (Duch.), *Agaricus campestris*, *Carthamus tinctorius* L., *Oryza sativa* L., *Raphanus sativus* L., *Cyphomandra betacea*, *Malachium aquaticum*, and *Zea mays* [18,20,62,64,68,76,77,78,79,80,81,82,83,84,85,86]. In addition, overexpression of *MsSNAT* increased endogenous melatonin level, and reduced ROS accumulation in transgenic *Arabidopsis* plants [19].

Melatonin scavenges the above ROS mainly through two pathways upon Cd stress. Antioxidant enzymes play key roles in melatonin-decreased ROS overproduction, such as APX, CAT, SOD, POD, GPX, GR, DHAR, and monodehydroascorbate reductase (MDHAR). Their functions are confirmed in above plant species. For example, exogenously applied with melatonin counterbalanced the H_2_O_2_ and MDA accumulation via enhancing APX, CAT, SOD, and POD activities under Cd stress [77]. Enzymes involved in the ascorbate-glutathione (AsA-GSH) cycle, such as DHAR, MDHAR and GR, were also involved in melatonin-mediated ROS balance in sunflower (*Carthamus tinctorius* L.) seedlings [80]. In addition, melatonin interacted with ROS by improving antioxidant levels, including GSH, AsA, and dehydroascorbate (DHA) [80]. Other studies reported melatonin also could increase proline, anthocyanins, flavonoid, and sugars contents in response to Cd-induced oxidative stress [18,64,77,79]. These impacts of melatonin on Cd-induced oxidative stress are summarized in Table 2. 

### 3.2. Melatonin Regulates Cadmium Uptake and Translocation

In general, Cd is taken up by plant roots from soil, then transported to shoots through the xylem and phloem, and eventually accumulated in grains [87]. Several processes regulate Cd accumulation, including Cd apoplastic influx, cell wall adsorption, cytoplasm across the membrane, xylem loading, vacuolar sequestration, and energy-driven transport in plants [88]. Natural resistance-associated macrophage protein (*NRAMP*) might be involved in several processes, such as uptake, intracellular transport, translocation, and metal detoxification in various plants [89,90]. Moreover, Cd is also transported through Zn, Fe, and Ca transporters, including Zn transporter proteins (ZRT)- and Fe-regulated transporter (IRT)-like protein (ZIP), yellow strip-like (YS1/YSL), and low-affinity calcium (Ca) transporter 1 (LCT1) [91]. ABC transport (PDR8), metal tolerance proteins (MTPs), cation diffusion facilitators (CDFs), and the P18-type metal transporter ATPase (HMAs) take part in Cd homeostasis [92,93,94]. Furthermore, GSH and its derivatives, phytochelatins (PCs), bound with Cd, and then transported Cd to vacuoles by ATP-binding cassette subfamily C proteins (ABCCs) [95,96]. HMA3 and CDF transporter family are also involved in the transfer of Cd–PCs complexes into the vacuole [97,98]. Other high-affinity chelators, including metallothioneins (MTs), organic acids, and amino acids play multiple roles in detoxification of Cd [99].

Recent studies have shown that melatonin regulates Cd homeostasis in plants. Exogenous application of melatonin reduced Cd contents in both roots and leaves of *Raphanus sativus* L. and *Brassica pekinensis* (Lour.) Rupr. plants [68,84]. Melatonin significantly decreased Cd contents in the leaves, but not in the roots of *Oryza sativa* L., *Carthamus tinctorius* L., and *Solanum lycopersicum* [61,76,80,81]. However, melatonin increased and decreased Cd contents in roots and shoots of *Malva parviflora*, respectively [18]. These results suggest that the effect of melatonin on translocation factor (Cd content of shoot/root) are different in the above various plants. Melatonin reduced the transcripts of metal transporter-related genes (iron-regulated transporter1 (*OsIRT1*), iron-regulated transporter2 (*OsIRT2*), heavy metal ATPase2 (*OsHMA2*), heavy metal ATPase3 (*OsHMA3*), natural resistance-associated macrophage protein1 (*OsNramp1*), natural resistance-associated macrophage protein5 (*OsNramp5*), and low-affinity cation transporter1 (*OsLCT1*) in leaves, but not in the roots of *Oryza sativa* L. under Cd stress [81]. Expression of *YSLs* and *HMAs* were down-regulated by melatonin, thereby reducing the Cd entering the roots of *Raphanus sativus* L. [84]. In addition, the Metallothionein 1 (*RsMT1*) gene was involved in melatonin-conferred Cd tolerance in transgenic tobacco [84]. In roots of *Brassica pekinensis* (Lour.) Rupr. plants, *IRT1* transcript was down-regulated significantly by melatonin application [68]. Then, Cd content was reduced in root tissues. These impacts of melatonin on Cd uptake and translocation are summarized in Table 3. Therefore, to characterize the biological roles of these metal transporter genes contributes to understanding the melatonin-mediated Cd homeostasis and detoxification.

### 3.3. Other Regulators Are Involved in Melatonin-Mediated Cd Tolerance

It has been widely reported that NO plays a crucial role in regulating various plant physiological processes [100]. Previous studies found that Cd treatment increased NO production, which promoted Cd accumulation by the *IRT1* up-regulation [101,102]. Exogenous melatonin alleviated Cd toxicity by reducing NO accumulation and *IRT1* expression in *Brassica pekinensis* (Lour.) Rupr. [68]. By contrast, melatonin triggered the endogenous NO, and enhanced Cd tolerance via the increase in the activities of antioxidant enzymes in wheat seedlings [20]. Moreover, melatonin can be nitrosated to NOMela by employing four nitrosating entities at the N atom of indole ring [46]. It was suggested that NOMela could release NO. That NO induces *S*-nitrosation is an important redox-based post-translational modification, which is involved in plant responses to abiotic stress [103,104]. Thus, complex interactions between melatonin and NO in Cd resistance should be further investigated. Another important signaling element, salicylic acid (SA), alleviated Cd toxicity by affecting Cd distribution, the antioxidant defense activities, and photosynthesis [105,106,107]. Amjadi et al. found that there was a possible synergic interaction between melatonin and SA by reducing Cd uptake and modulating the ascorbate–glutathione cycle and glyoxalase system [80].

## 4. A Possible Role for H_2_S in Melatonin-Mediated Tolerance against Cd Stress

Acting as a signaling molecule, NO interacts with other molecules (H_2_O_2_, CO, and H_2_S) to mediate plant growth and development, as well as abiotic stress responses [100]. Among the molecules, H_2_S is also involved in almost all physiological plant processes [27,100]. To date, there is considerable research on the role of NO in melatonin-modulated plant abiotic stress tolerance. However, the functions of H_2_S have been largely unknown. It will become a research hotspot to contribute to precise analysis of the collaboration between H_2_S and melatonin, and provide deeper insight into melatonin-mitigated signaling mechanisms.

### 4.1. H_2_S Action in Plant Tolerance against Cd Stress

**Figure 3:**
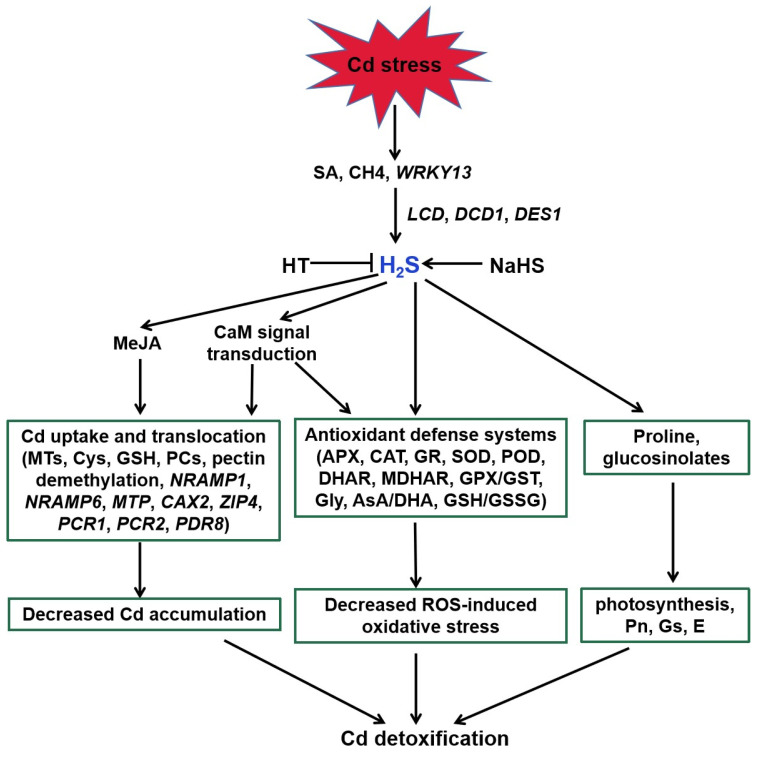
Function of H_2_S in plant responses to Cd stress. SA, CH_4_, and *WRKY13* are involved in Cd-induced H_2_S generation. H_2_S enhances the antioxidant defense systems to decrease the ROS accumulation, regulates the transcripts of genes related to Cd uptake and translocation to reduce the Cd accumulation, and increases proline and glucosinolates in response to Cd stress in plants. MeJA and Ca participate in the above regulatory pathways. SA, salicylic acid; CH4, methane; HT, hypotaurine; LCD, L-cysteine desulfhydrase; DCD, D-cysteine desulfhydrase; *DES1*, L-cysteine desulfhydrase 1; MeJA, methyl jasmonate; CaM, calmodulin; *NRAMP1*, natural resistance-associated macrophage protein1; *NRAMP6*, natural resistance-associated macrophage protein6; *MTP*, metal tolerance protein; *CAX2*, vacuolar cation/proton exchanger2; *ZIP4*, zinc-iron permease4; *PCR1*, plant cadmium resistance1; *PCR2*, plant cadmium resistance2; *PDR8*, pleiotropic drug resistance8.

H_2_S acts as a signaling molecule in modifying various metabolic processes in plants, especially Cd stress (Figure 3, [27]). Endogenous H_2_S production was induced via expression of *LCD*, *DCD*, and *DES1* under Cd stress [108,109,110]. SA, methane (CH_4_), and WRKY DNA-binding protein 13 (*WRKY13*) transcription factor were suggested to be involved in the above process [30,111,112]. H_2_S regulated the activities of key enzymes and AsA-GSH cycle involved in ROS homeostasis to alleviate Cd-induced oxidative stress [113,114,115,116,117,118,119,120]. For example, H_2_S enhanced the activities of antioxidant enzymes, such as POD, CAT, APX, and SOD, and thereby decreased ROS accumulation [120]. Similarly, it also obviously increased AsA and GSH and the redox status (AsA/DHA and GSH/GSSG) levels to improve rice Cd resistance [114,116].

Increasing evidence demonstrates that H_2_S also regulates Cd uptake and translocation in plants [30,117,119,121]. H_2_S enhanced the expression of genes encoding metallothionein (MTs) and phytochelatin (PCS) in *Arabidopsis* roots [117]. Therefore, H_2_S increased the metal chelators synthesis, contributing to Cd detoxification by binding the trace metal. In addition to enhancing the above genes expression, the protective effect of H_2_S was attributed to a decrease in Cd accumulation associated with the expression of Cd transporter genes, such as *PCR1*, *PCR2*, and *PDR8* [30]. Exogenous application of NaHS weakened the expression of *NRAMP1* and *NRAMP6* genes, and intensified the expression of Cd homeostasis-related genes (*CAX2* and *ZIP4*) to enhance Cd tolerance in foxtail millet [122].

**Figure 4:**
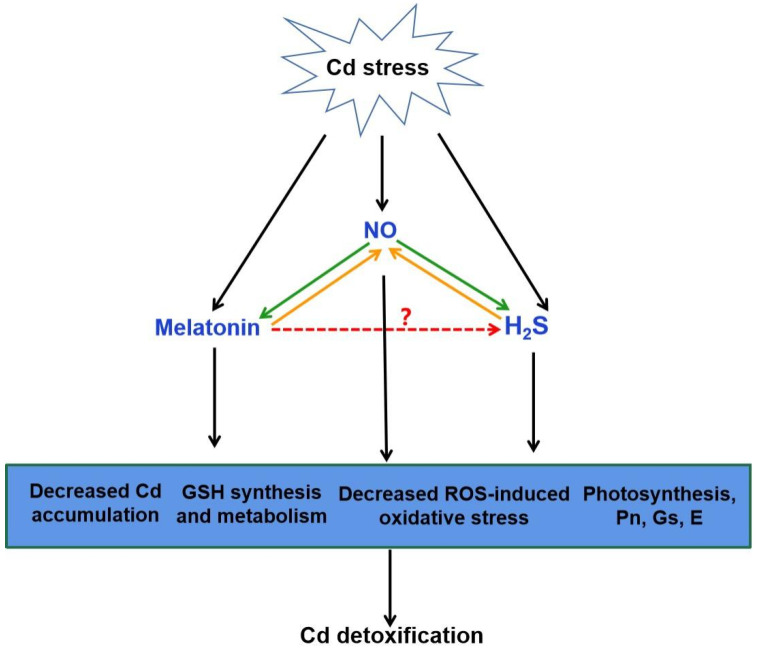
The possible role of H_2_S in melatonin-mediated Cd detoxification. NO generation can be induced by Cd stress. Increasing evidence showed that melatonin and H_2_S act as the downstream of NO in the responses to Cd stress, respectively (green arrow). It is also suggested that NO acts as a downstream of melatonin or H_2_S to improve Cd tolerance (orange arrow). The combination of melatonin, NO and H_2_S might be responsible for melatonin-triggered signal transduction in plant Cd tolerance via the decreased Cd accumulation, GSH synthesis and metabolism, decreased ROS-induced oxidative stress and improved photosynthesis. Red arrow, yet largely unknown. Cd, cadmium; NO, nitric oxide; H_2_S, hydrogen sulfide; GSH, glutathione; ROS, reactive oxygen species; Pn, photosynthesis rate; Gs, stomatal conductance; E, transpiration.

A number of studies address that H_2_S can interact with other signaling molecules, such as SA, proline, MeJA, Ca, and NO during the responses of plants to Cd stress (Figure 3 and Figure 4; [111,122,123]). H_2_S acted as a downstream molecule of SA-transmitted signals to regulate Cd tolerance in *Arabidopsis* [111]. The endogenous production of proline and MeJA enhanced by H_2_S donor NaHS responded significantly to Cd stress in foxtail millet [122,123]. H_2_S also improved CaM gene expression and controlled the combination of Ca^2+^ and CaM, which act as signal transducers [33].

There exists a complicated and synergistic relationship between H_2_S and NO in response to Cd stress in plants (Figure 4; [115,118,124,125]). Exogenous NO and H_2_S application increased the Cd tolerance in plants [115,124,126]. Subsequent pharmacological experiments proved that H_2_S donor NaHS triggered NO production, which might act as a signal for alleviation of Cd-induced oxidative damage in alfalfa seedling roots [124]. Nevertheless, H_2_S production activated by NO is essential in Cd stress response of bermudagrass [115]. As a second messenger, Ca acted both upstream and downstream of NO signal, and crosstalk of Ca and NO regulated the cysteine and H_2_S to mitigate Cd toxicity in Vigna radiata [126]. Moreover, application of sodium nitroprusside (SNP), the donor of NO, increased H_2_S generation, and thus enhanced Cd stress tolerance in wheat [118]. However, this protective effect was reversed by hypotaurine (HT), the scavenger of H_2_S [118]. These results suggested that H_2_S and NO can function in a coordinated way under certain signaling cascades in plants under Cd stress.

### 4.2. Crosstalk of Melatonin and H_2_S in Plants

The interaction between melatonin and H_2_S plays a beneficial role in abiotic stress response [32]. Exogenous melatonin regulated the endogenous H_2_S homeostasis by modulating the L-DES activity in salt-stressed tomato cotyledons [31]. Moreover, an endogenous H_2_S-dependent pathway was involved in melatonin-mediated salt stress tolerance in tomato seedling roots [34]. Synergistic effects of melatonin and H_2_S regulated K^+^/Na^+^ homeostasis, and reduced excessive accumulation of ROS by enhancing the activity of antioxidant enzymes. Inhibition of H_2_S by HT reversed the melatonin-modulated heat tolerance by inhibiting photosynthesis, carbohydrate metabolism, and the activity of antioxidant enzymes in wheat [36]. Recent investigation has revealed that melatonin-induced pepper tolerance to iron deficiency and salt stress was dependent on H_2_S and NO [118]. It was further confirmed that H_2_S and NO jointly participated in melatonin-mitigated salt tolerance in cucumber [35]. Thus, these results postulate that H_2_S might act as a downstream signaling molecule of melatonin. Combined with the roles of H_2_S and melatonin in alleviating Cd stress, it is easy to speculate that H_2_S might be involved in melatonin-mediated Cd tolerance in plants (Figure 4).

As mentioned above in Section 3, GSH plays a critical role in plant Cd tolerance. It is synthesized from glutamate, cysteine and glycine by *γ*-glutamyl cysteine synthetase (*γ*-ECS, encoded by *GSH1*/*ECS* gene) and glutathione synthetase (GS, encoded by *GSH2*/*GS* gene) [127]. The catalysis of GSH1 is the rate-limiting step of GSH biosynthesis [128]. Cd stress induced the transcripts of *GSH1* and *GSH2* in *Arabidopsis*, as well as *ECS* and *GS* in *Medicago sativa* [114,129,130,131]. It was suggested that H_2_S could be quickly incorporated into cysteine and subsequently into GSH [132]. Application of NaHS re-established (h)GSH homeostasis by further strengthening the up-regulation of *ECS* and *GS* genes [114]. Similar results were also found in strawberry and cucumber plants [133,134]. Interestingly, exogenous melatonin also increased the GSH content by inducing the transcript levels of *SlGSH1* in tomato [75]. Hence, there might be a certain connection between H_2_S and melatonin in regulating the GSH homeostasis at the transcriptional regulatory pathway. This will provide an interesting direction for further research on the complex interactions between melatonin and H_2_S in improving Cd tolerance in plants.

## 5. Conclusions and Future Prospects

Recent studies have strongly indicated that melatonin, a multifunctional molecule, regulates Cd tolerance in plants. To further promote related research in plant Cd tolerance, this review summarizes the regulatory roles and mechanisms of melatonin in response to Cd stress. Melatonin reduces Cd damage mainly through re-establishing the redox homeostasis and decreasing Cd accumulation, but its underlying mechanisms remain to be determined. Intriguingly, melatonin is suggested to be a phytohormone due to the identification of the putative receptor CAND2/PMTR1 [135], although there is still a debate on whether it is a *bona fide* receptor for melatonin [136]. More importantly, more receptor gene(s) should be characterized, which will be critical for precisely understanding the signal transduction pathway of melatonin in plants in response to Cd stress.

Currently, as a signal molecule, the role of NO has been revealed in melatonin-mediated Cd tolerance, likewise H_2_S plays a key messenger in plant resistance to Cd stress. That the effects of H_2_S have been less explored has prevented precise analysis of the collaboration of H_2_S and melatonin. Recently, we presented the underlying mechanisms of H_2_S action and its multifaceted roles in plant stress responses [137]. Hence, it would be interesting to fully evaluate the effects of H_2_S-based signaling on regulating melatonin-induced Cd tolerance. For directions of future research, biochemical and genetic characterization of H_2_S-producing proteins and persulfidation signaling is needed and will shed more light on the integration of H_2_S and melatonin signaling during Cd stress.

### 5.1. Pharmacological, Genetic and ‘Omics’ Approach to Understand the Crosstalk of H_2_S–Melatonin during Cd Stress

Various pharmacological, enzyme activity, and gene expression investigations revealed the crosstalk of H_2_S–melatonin in response to salt and heat stress in tomato and cucumber [34,35,36]. Exogenous melatonin induced the H_2_S generation by activating the L-cysteine desulfhydrase (L-CDes) activity, which was encoded by *LCD*, *DCD*, and *DES1* [31,35]. Then, the interaction of H_2_S and melatonin enhanced the antioxidant defense, and regulated carbohydrate metabolism and ion homeostasis [34,35,36,118]. Similar pharmacological experiments with an effective concentration range of 1–200 μM in exogenous melatonin, 10–100 μM in 4-Chloro-DL-phenylalanine (p-CPA, melatonin synthesis inhibitor), 10–100 μM in hypotaurine (HT, H_2_S inhibitor), and 10–100 μM in NaHS (H_2_S donor) could be used to investigate the crosstalk of H_2_S–melatonin during Cd stress. Furthermore, genetic modifications with altering melatonin and H_2_S levels, such as *snat*, *comt*, *lcd*, *dcd*, and *des1* mutants, should be used to explore their possible roles.

H_2_S plays a critical signal mediator in plants in response to Cd stress [111,112,115]. However, there is still an urgent need to elucidate the interactions of H_2_S with other signaling molecules in melatonin-mediated Cd tolerance. With the advent of transcriptomic and proteomic analysis, scientists shall reveal the intrinsic regulatory mechanisms of melatonin and H_2_S interaction on the regulation of various biological processes. For example, the expression of genes and proteins related to GSH synthesis and metabolism and redox homoeostasis, as well as the hormone biosynthesis pathways, might be used to establish a model system to decipher their signaling interaction.

### 5.2. The Potential Role of Persulfidation Driven by H_2_S in Melatonin-Mediated Cd Tolerance

Recently, it was found that H_2_S-mediated post-translational modification (PTM, persulfidation) of protein cysteine residues (RSSH) is an important mechanism in plants to adapt to external environments [27,32]. Protein persulfidation cause various changes in structures, activities, as well as the subcellular localizations of the candidate proteins [138,139]. These proteins are mainly involved in plant growth and development, abiotic stress responses, and carbon/nitrogen metabolism [138]. For example, H_2_S production regulated the persulfidation of NADPH oxidase RBOHD at Cys825 and Cys890, leading to improving the ability to produce H_2_O_2_ signal [140]. It also led to the persulfidation of ABSCISIC ACID INSENSITIVE 4 (ABI4) at Cys250, and persulfidation of SnRK2.6, contributing to reveal the function of H_2_S in the complex signal-transduction system [141,142,143]. By contrast, the residue Cys32 of APX could be persulfidated, thereby enhancing its activity [144]. Therefore, the persulfidation might become a promising direction to investigate the roles of H_2_S in melatonin-mediated Cd tolerance in plants. To conclude, the progresses in the various physiological and molecular mechanisms regulated by melatonin are not enough, and future studies along with the above lines should be used to unveil the regulatory mechanism of melatonin and H_2_S signaling pathways in plant Cd tolerance.

## Figures and Tables

**Table 1:** Summary table explaining the effect of Cd on genes related to melatonin metabolic pathway.

Plant Species	Cd Stress and Duration	Impact on Genes Related to Melatonin Metabolic Pathway	References
*Solanum lycopersicum*	100 μM Cd^2+^ for 15 d	*TDC*, *T5H*, *COMT* genes (leaves)	[22]
*Oryza sativa* L.	500 μM Cd^2+^ for 3 d	*TDC1*, *TDC3*, *SNAT1*, *SNAT2*, *ASMT*, *COMT*, *M2H*, *M3H* genes (seedlings)	[23]
*Triticum aestivum* L.	200 μM Cd^2+^ for 1 d	*ASMT*, *COMT*, *TDC* genes (root and shoot)	[62]
*Nicotiana tabacum* L.	10 mg/kg Cd^2+^ for 1, 4, and 7 d	*SNAT1* gene (leaves)	[63]
*Agaricus campestris*	2, 5, or 8 μM Cd^2+^ for 5 d	*TDC*, *T5H*, *SNAT*, *ASMT*, *COMT* genes	[64]
*Oryza sativa* L.	200 μM Cd^2+^ for 6, 12, 24, 72 h	*SNAT*, *ASMT*, *COMT*, *TDC*, *T5H* genes (leaves)	[65,67]
*Arabidopsis thaliana*	300 μM Cd^2+^ for 2, 3, 4 d	*SNAT*, *COMT* genes (leaves)	[66]

*TDC1*, tryptophan decarboxylase1; *T5H*, tryptamine 5-hydroxylase; *COMT*, caffeic acid O-methyltransferase; *SNAT1*, serotonin N-acetyltransferase1; *SNAT2*, serotonin N-acetyltransferase2; *ASMT*, N-acetylserotonin-O-methyltransferase; *M2H*, melatonin 2-hydroxylase; *M3H*, melatonin 3-hydroxylase.

**Table 2:** Summary table explaining the impacts of melatonin on Cd-induced oxidative stress.

Plant Names	Treatments	Impact on Oxidative Stress Markers and Antioxidative Defense Systems	References
*Nicotiana tabacum* L.	0, 25, 50, 100, and 250 μM melatonin; 100 μM Cd^2+^ for 7 d	H_2_O_2_, O_2_·^−^; APX, SOD, CAT (leaves)	[6]
*Malva parviflora*	0, 15, 50, and 100 μM melatonin; 50 μM Cd^2+^ for 8 d	H_2_O_2_, MDA, SOD, CAT, GPX, PAL, flavonoid, anthocyanins (shoots)	[18]
*Medicago sativa* L.	0, 10, 50, and 200 μM melatonin; 100 μM Cd^2+^ for 1, 3 d	H_2_O_2_, O_2_·^−^; SOD (roots)	[19]
*Triticum aestivum* L.	0, 50, and 100 μM melatonin; 100 μM Cd^2+^ for 28 d	H_2_O_2_, MDA; SOD, CAT, POD (leaves)	[20]
*Triticum aestivum* L.	0, 50, and 100 μM melatonin; 100 μM Cd^2+^ for 12, 24, 48 h	H_2_O_2_; APX, SOD, CAT, POD, GSH/GSSG (leaves and roots)	[62]
*Agaricus campestris*	0, 50, 100, and 200 μM melatonin; 2, 5, and 8 μM Cd^2+^ for 5 d	H_2_O_2_, MDA; SOD, CAT, POD, APX, GR, proline, sugars	[64]
*Solanum lycopersicum*	0, 25, 50, 100, 250, and 500 μM melatonin; 100 μM Cd^2+^ for 14 d	H_2_O_2_, MDA, O_2_·^−^; SOD, CAT, GR, POD, APX (leaves)	[75]
*Solanum lycopersicum*	100 μM melatonin; 100 μM Cd^2+^ for 15 d	H_2_O_2_; APX, SOD, CAT, POD (leaves and roots)	[76]
*Brassica napus* L.	0, 50, and 100 μM melatonin; 20 μM Cd^2+^ for 5 d	H_2_O_2_, MDA; APX, SOD, CAT, POD, proline, anthocyanins (seedlings)	[77]
*Catharanthus roseus* (L.)	100 μM melatonin; 0, 50, 100, and 200 mg Cd kg^−1^ soil for 30 d	H_2_O_2_; CAT, POD (leaves)	[78]
*Fragaria x ananassa* (Duch.)	0, 10, 50, 100, 150, and 200 μM melatonin; 300 mL of 1 mmol·L^−1^ Cd^2+^ for 5, 10 d	MDA; SOD, CAT, POD, APX, soluble protein, anthocyanins (leaves and roots)	[79]
*Carthamus tinctorius* L.	100 μM melatonin; 100 μM Cd^2+^ for 21 d	H_2_O_2_, MDA, LOX; ASA, DHA, GSH, GSSG, SOD, APX, DHAR, CAT, GR, MDHAR, Gly (leaves)	[80]
*Oryza sativa* L.	0, 50, 100, and 200 μM melatonin; 100 μM Cd^2+^ for 10 d	H_2_O_2_, MDA; SOD, CAT, POD (leaves and roots)	[81]
*Zea mays*	200 μM melatonin; 150 μM Cd^2+^ for 3 d	MDA; SOD, CAT, POD (root, stem, and leaf)	[82]
*Oryza sativa* L.	100 μM melatonin	MDA; SOD, CAT, POD (shoots)	[83]
*Raphanus sativus* L.	0, 10, 25, 50, 100, and 200 μM melatonin; 50 μM Cd^2+^ for 24 h	SOD, CAT, POD, APX, GR (roots and shoots)	[84]
*Malachium aquaticum,* *Galinsoga parviflora*	0, 50, 100, 150, and 200 μM melatonin; 10 mg/L Cd for 40 d	SOD, POD, CAT (leaves)	[85]
*Cyphomandra betacea*	0, 50, 100, 150, and 200 μM melatonin; 10 mg/L Cd for 40 d	SOD, POD, CAT (leaves)	[86]

H_2_O_2_, hydrogen peroxide; MDA, malondialdehyde; O_2_**·**^−^, superoxide anion; APX, ascorbate peroxidase; SOD, superoxide dismutase; CAT, catalase; GPX, glutathione peroxidase; PAL, phenylalanine ammonia-lyase; POD, guaiacol peroxidase; GSH/GSSG, reduced (GSH)/oxidized (GSSG) glutathione; GR, glutathione reductase; LOX, lipoxygenase; ASA, ascorbate; DHA, dehydroascorbate; DHAR, dehydroascorbate reductase; MDHAR, monodehydroascorbate reductase; Gly, glycine.

**Table 3:** Summary table explaining the impacts of melatonin on Cd uptake and translocation.

Plant Names	Treatments	Impact on Cd in Subcellular Compartment	References
*Nicotiana tabacum* L.	0, 25, 50, 100, and 250 μM melatonin; 100 μM Cd^2+^ for 7 d	Cd content in leaves; H^+^-ATPase activity, *IRT1*, *IRT2*, *Nramp1*, *HMA2*, *HMA3*, *HMA4*	[6]
*Malva parviflora*	0, 15, 50, and 100 μM melatonin; 50 μM Cd^2+^ for 8 d	Cd content in shoots	[18]
*Medicago sativa* L. *Arabidopsis*	0, 10, 50, and 200 μM melatonin; 100 μM Cd^2+^ for 1, 3 d	Cd content in leaves; *PCR2*, *Nramp6*, *PDR8*, *HMA4*	[19]
*Solanum lycopersicum*	1 μM melatonin; 100 μM Cd^2+^ for 15 d	Cd content in leaves; GSH and PCs	[61]
*Brassica pekinensis* (Lour.) *Rupr.*	100 μM melatonin; 20 μM Cd^2+^ for 24 h	Cd contents in roots and leaves; *IRT1/2*	[68]
*Solanum lycopersicum*	0, 25, 50, 100, 250, and 500 μM melatonin; 100 μM Cd^2+^ for 14 d	Cd content in leaves; H^+^-ATPase activity, GSH and PCs; *SlGSH1*, *SlPCS*, *SlMT2*, and *SlABC1*	[75]
*Solanum lycopersicum*	100 μM melatonin; 100 μM Cd^2+^ for 15 d	Cd content in leaves; Cys, γ-glutamyl cysteine, GSH and PCs	[76]
*Brassica napus* L.	0, 50, and 100 μM melatonin; 20 μM Cd^2+^ for 5 d	Cd content; H^+^-ATPase activity	[77]
*Carthamus tinctorius* L.	100 μM melatonin; 100 μM Cd^2+^ for 21 d	Cd content in roots, stems and leaves; PCs	[80]
*Oryza sativa* L.	0, 50, 100, and 200 μM melatonin; 100 μM Cd^2+^ for 10 d	Cd content in leaves; *OsIRT1*, *OsIRT2*, *OsHMA2*, *OsHMA3*, *OsNramp1*, *OsNramp5*, and *OsLCT1*	[81]
*Oryza sativa* L.	100 μM melatonin	Cd content in roots and shoots; *Nramp1*, *Nramp5*, *IRT1*, *IRT2*, *HMA2*, *HMA3*	[83]
*Raphanus sativus* L.	0, 10, 25, 50, 100, and 200 μM melatonin; 50 μM Cd^2+^ for 24 h	Cd content in roots and leaves; *PCS*; *MT*, *CAX4*, *ZIP12*, *HMA4*, *YSL2*, *YSL7*	[84]
*Malachium aquaticum,* *Galinsoga parviflora*	0, 50, 100, 150, and 200 μM melatonin; 10 mg/L Cd for 40 d	Cd content in leaves	[85]
*Cyphomandra betacea*	0, 50, 100, 150, and 200 μM melatonin; 10 mg/L Cd for 40 d	Cd contents in stems, leaves, and shoots	[86]

*IRT1*, iron-regulated transporter1; *IRT2*, iron-regulated transporter2; *Nramp1*, natural resistance-associated macrophage protein1; *Nramp5*, natural resistance-associated macrophage protein5; *HMA2*, heavy metal ATPase2; *HMA3*, heavy metal ATPase3; *HMA4*, heavy metal ATPase4; *PCR2*, plant cadmium resistance2; *PDR8*, pleiotropic drug resistance8; *GSH1*, glutamate-cysteine ligase; *PCS*, phytochelatin synthase activity; *MT*, metallothionein; *ABC1*, ATP-binding cassette transporter1; *LCT1*, low-affinity cation transporter; *CAX4*, vacuolar cation/proton exchanger4; *ZIP12*, zinc-iron permease12; *YSL2*, yellow stripe-like transporter2; *YSL7*, yellow stripe-like transporter7.

## Data Availability

Not applicable.
